# Inefficient cationic lipid-mediated siRNA and antisense oligonucleotide transfer to airway epithelial cells in vivo

**DOI:** 10.1186/1465-9921-7-26

**Published:** 2006-02-15

**Authors:** Uta Griesenbach, Chris Kitson, Sara Escudero Garcia, Raymond Farley, Charanjit Singh, Luci Somerton, Hazel Painter, Rbecca L Smith, Deborah R Gill, Stephen C Hyde, Yu-Hua Chow, Jim Hu, Mike Gray, Mark Edbrooke, Varrie Ogilvie, Gordon MacGregor, Ronald K Scheule, Seng H Cheng, Natasha J Caplen, Eric WFW Alton

**Affiliations:** 1 Department of Gene Therapy, Faculty of Medicine at the National Heart and Lung Institute, Imperial College, London, UK; 2 GlaxoSmithKline, UK; 3 Gene Medicine Research Group, Nuffield Department of Clinical Laboratory Sciences, John Radcliffe Hospital, University of Oxford, UK; 4 Programme in Lung Biology Research, Hospital for Sick Children and Department of Laboratory Medicine and Pathobiology, University of Toronto; 5 Institute for Cell and Molecular Biosciences, University Medical School, Newcastle, UK; 6 Medical Genetics Section, University of Edinburgh, Edinburgh, UK; 7 Genzyme Corporation, USA; 8 Medical Genetics Branch, National Human Genome Research Institute, National Institutes of Health, Bethesda, MD 20892; 9 Gene Silencing Section, National Cancer Institute, National Institutes of Health, Bethesda, MD 20892; 10 UK Cystic Fibrosis Gene Therapy Consortium

## Abstract

**Background:**

The cationic lipid Genzyme lipid (GL) 67 is the current "gold-standard" for *in vivo *lung gene transfer. Here, we assessed, if GL67 mediated uptake of siRNAs and asODNs into airway epithelium *in vivo*.

**Methods:**

Anti-lacZ and ENaC (epithelial sodium channel) siRNA and asODN were complexed to GL67 and administered to the mouse airway epithelium *in vivo *Transfection efficiency and efficacy were assessed using real-time RT-PCR as well as through protein expression and functional studies. In parallel *in vitro *experiments were carried out to select the most efficient oligonucleotides.

**Results:**

*In vitro*, GL67 efficiently complexed asODNs and siRNAs, and both were stable in exhaled breath condensate. Importantly, during *in vitro *selection of functional siRNA and asODN we noted that asODNs accumulated rapidly in the nuclei of transfected cells, whereas siRNAs remained in the cytoplasm, a pattern consistent with their presumed site of action. Following *in vivo *lung transfection siRNAs were only visible in alveolar macrophages, whereas asODN also transfected alveolar epithelial cells, but no significant uptake into conducting airway epithelial cells was seen. SiRNAs and asODNs targeted to β-galactosidase reduced βgal mRNA levels in the airway epithelium of K18-lacZ mice by 30% and 60%, respectively. However, this was insufficient to reduce protein expression. In an attempt to increase transfection efficiency of the airway epithelium, we increased contact time of siRNA and asODN using the *in vivo *mouse nose model. Although highly variable and inefficient, transfection of airway epithelium with asODN, but not siRNA, was now seen. As asODNs more effectively transfected nasal airway epithelial cells, we assessed the effect of asODN against ENaC, a potential therapeutic target in cystic fibrosis; no decrease in ENaC mRNA levels or function was detected.

**Conclusion:**

This study suggests that although siRNAs and asODNs can be developed to inhibit gene expression in culture systems and certain organs *in vivo*, barriers to nucleic acid transfer in airway epithelial cells seen with large DNA molecules may also affect the efficiency of *in vivo *uptake of small nucleic acid molecules.

## Background

The inhibition of gene expression mediated by antisense oligonucleotides (asODN) has a long history. The first asODN-based drug (Vitravene) for the treatment of cytomegalovirus (CMV)-induced retinitis in AIDS patients has been approved [1], and several phase I, II and III trials for the treatment of cancer and a variety of inflammatory conditions are currently ongoing. AsODNs have also been considered for treatment of a variety of lung diseases including asthma and other pulmonary inflammatory diseases and have shown some efficacy in pre-clinical models after nebulisation, intratracheal injection, intravenous or intraperitoneal administration. Phase I trials using asODN against the adenosine A(1) receptor have been carried out in asthmatics and shown to be safe but phase IIa trials did not demonstrate efficacy in patients using inhaled steroids. [2] Effective asODN can be generated against intronic and splice-site sequences [3,4], implying that asODN function mainly in the nucleus, where they bind to mRNA target sequence specifically by forming Watson-Crick base pairs. The mRNA/asODN hybrid is subsequently recognised by RNase H, which leads to degradation of the mRNA target.

More recently RNA interference (RNAi), using short (<30 bp) double-stranded RNA molecules termed siRNAs, has emerged as an alternative gene silencing strategy. RNAi was first identified in plants and invertebrates, but more recently also in mammalian cells. Since the studies in mammalian cells [5,6] a large number of publications now document the use of RNAi in cell culture-based systems and the power of RNAi for drug validation and studies of enzyme pathways is well recognized. The use of RNAi as a therapeutic approach is in its infancy, but several organs, including liver, eye, lung, brain, skeletal muscle, as well as tumours, have been targeted successfully *in vivo*.

Antisense or RNAi-mediated gene silencing may provide novel opportunities for the treatment of cystic fibrosis (CF). CF is caused by mutations in the cystic fibrosis transmembrane conductance regulator gene (*CFTR*) and affects many organs, but most morbidity and mortality relates to chronic inflammation and bacterial colonisation of the lung. The *CFTR *gene encodes a chloride channel in the apical membrane of epithelial cells, and in CF patients chloride secretion through CFTR is reduced or absent. This, coupled with increased sodium absorption through the epithelial sodium channel (ENaC), leads to abnormal water transport across the epithelium and accumulation of sticky, dehydrated mucus, which in turn leads to chronic bacterial colonisation and inflammation (reviewed in [7]). Similar to CFTR, ENaC is expressed in airway surface epithelium and glands [8]. Normally CFTR inhibits ENaC-mediated sodium transport, although the mechanism is not completely understood [9,10]. In CF this inhibition is lost, resulting in increased sodium and water absorption. Down-regulation of ENaC expression, or inhibition of its function, may therefore attenuate CF lung disease. The latter has proved difficult [11] because of the short half-life of amiloride, and potential renal side effects of longer acting inhibitors. With regards to the former, ENaC consists of 3 separate subunits (α, β and γ). Jain *et al *generated asODN against all three subunits, and demonstrated that only asODN against the α subunit significantly decreased the density of ENaC channels [12].

Transfection of differentiated airway epithelial cells with plasmid DNA (pDNA), which is generally >5000 bp, is inefficient, and the extra- and intracellular barriers to airway gene transfer have been identified over the last decade (reviewed in [13]). Here, we assessed, whether smaller nucleic acids such as asODNs (20 bases) or siRNA (20–22 base pairs) can more readily overcome these barriers, and thereby provide an alternative approach for the treatment of CF lung disease.

## Material and methods

### SiRNA, asODN and *in vitro *assessment of lipid complexation

The asDNA used in this study were designed using a proprietary algorithm (GlaxoSmithKline, Stevenage, UK). The oligonucleotides were 'gapmers' and consisted of 2'O-methyl RNA (5 residues), phosporothioate DNA (10 residues), 2'O-methyl RNA (5 residues), and were synthesised by Proligo (Hamburg, Germany) [14]. Synthetic siRNAs (CAT, GFP, LacZ) were synthesized by Xeragon using 29-*O*-(tri-isopropyl) silyloxymethyl chemistry (Qiagen Inc. Germantown MD, USA) as previously described [5]. Synthetic siRNAs corresponding to ENaC were obtained from Dharmacon Inc (Chicago, IL, USA).

SiRNA and asODN (1.6 mg/ml = 4.8 mM) were complexed to Genzyme Lipid 67 (GL67) at different molar ratios (lipid:DNA) in a total volume of 100 ul as previously described [13]. Following incubation, 8 μg siRNA and asDNA from each reaction were separated on an agarose gel (0.4%) to resolve lipid-complexed and free nucleotides. Controls included a standard plasmid (6 kb) complexed in a similar manner. At least two independent reactions were carried out for each condition and representative images are shown. Particle size was determined by dymanmic laser light scatter using a Coulter N4SD submicron particle analyser (Hialeah, USA).

### Stability in exhaled breath condensate (EBC)

CF patients were recruited through the Adult Cystic Fibrosis Service at the Western General Hospital in Edinburgh. Non-CF control subjects were recruited from staff at the Western General Hospital. Written informed consent was obtained and the study was approved by the Lothian Health Ethics Committee. Prior to collection of EBC, subjects rinsed their mouths with water. Subjects then breathed through a Jaeger Ecoscreen EBC collection device (Jaeger, Hoechberg, Germany) for 5 minutes. This allows subjects to perform tidal breathing through a two-way valve mechanism while trapping saliva. 500–1000 μl of EBC were collected from each individual. To determine the stability of asDNA or siRNA in fresh EBC, equal volumes of nucleic acid (diluted to 500 ng/μl in nuclease-free H_2_0) and EBC from either a CF patients (n = 2) or healthy individuals (n = 2) were incubated together for 1, 5, 30 and 60 minutes at 37°C. As positive and negative controls, each nucleic acid was incubated with either nuclease-free H_2_0, 200 ng RNase A (QIAGEN) or 2 units DNase 1 (Sigma), as appropriate, for 1, 5, 30 and 60 minutes at 37°C. After incubation, the samples were placed on ice and immediately characterised using the Agilent 2100 Bioanalyser microfluidics system (Agilent Technologies UK Ltd, Stockport, UK). For this, 1 μl of denatured sample was loaded onto a primed RNA 6000 chip and the integrity of each nucleic acid was determined from the digital output data.

### Cell culture based transfection

For cell culture based pre-screening of siRNAs approximately 2 × 10^5 ^NIH-3T3 cells stably expressing β-galactosidase (generated through retroviral transduction of an ecotropic retroviral vector carrying *LacZ *and G418 slection for neomycin resistance) were transfected with 2 μg of siRNA complexed with 10 μg Lipofectamine 2000 (Life Technologies, Gaithersburg, MD) in 12-well plates. SiRNA-lipid complexes were formed in unsupplemented medium (DMEM), and added directly to cells after approximately 15 minutes. Four hours after addition of the siRNA-lipoplex, DMEM plus 20% foetal bovine serum was added. Cells were harvested 72–96 hours after transfection and assayed for β-galactosidase protein expression

For cell culture based pre-screening of asDNA, NIH-3T3-lacZ cells were plated at 2 × 10^4 ^cells per well in a 96-well plate, 18 hours prior to transfection. Liposome complexes were made up as follows: for each well, 10 μl of 10× final concentration asDNA (100 or 200 nM) were diluted in OptiMem (Invitrogen, Paisley, UK) from a 100 M stock in water. This was mixed with 5 μg Lipofectamine 2000 (Invitrogen, UK) in 10 μl OptiMem, and incubated for 15 min at room temperature. Complexes were then diluted to a total volume of 100 μl in OptiMem and added directly to cells after washing with OptiMem. Forty-eight hours after transfection, cells were lysed and β-gal protein and total protein assayed using the β-gal reporter kit (Roche, Welwyn, UK) and the Coomassie Plus Protein Assay kit (Per-Bio, Cramlington, UK) according to manufacturer's recommendations. M1 cells (murine kidney epithelium) (ATCC) were grown to 70% confluency in 6-well-plates and transfected with ENaC siRNA or asDNA (100 nM or 200 nM) complexed to Lipofectamine 2000 (5 μg lipid/ml,) as described above. Forty-eight hours after transfection cells were harvested and total RNA prepared and quantitative RT-PCR carried out as described below.

To determine transfection efficiency and intracellular distribution, semi-confluent M1 cells were transfected in 8-well chamber slides with lipid-complexed FITC-labelled siRNA, asDNA (final concentration 100 nM), lipid only or left untransfected (VWR, Leics, UK). Transfection reagents and volumes were scaled down according to surface area (well in 6-well plate: 9.4 cm^2^, well in 8-well chamber slide 0.32 cm^2^). At different time-points after transfection (1, 15, 30, 60 min, 2, 4, 6, 8, 18 and 24 hours) cells were washed in PBS, fixed in 4% paraformaldehyde for 10 min, washed again in PBS, stained with DAPI (1 μg/ml) for 15 min and mounted with Vectashield (Invitrogen, Paisley, UK). Confocal microscopy was carried at an original magnification of 40×. Two independent experiments were carried out with n = 2 wells per condition. A minimum of 3 fields of view were analysed per well. Representative images are shown.

### *In vivo *transfection of lungs

All animal studies were approved by the UK Home Office and Imperial College London. Flourescently (FITC)-labelled asODN or siRNA (160 μg/mouse in 100 μl, the maximum total volume allowed for lung instillations) were complexed to GL67 at a 0.25:1 molar ratio (lipid:DNA) controls received lipid only. BALB/C mice (female 6–10 weeks, n = 3/group) were anaesthetised with metophane (Medical Developments Australia Pty Ltd, Springvale, Australia) and the liposome complexes were placed as a single bolus into the nasal cavity and the solution rapidly sniffed into the lungs. One and 24 hours after transfection animals were culled, the trachea exposed and the lungs inflated with 4% paraformaldehyde (pH 7.3) using a catheter (20 gauge, Ohmeda, Sweden) inserted into the trachea. The lungs were than removed *en bloc *and placed into 4% paraformaldehyde for a further 12–18 hours. The tissues were processed and paraffin-embedded using standard procedures and 5 μm sections cut (at least 5/mouse approximately 50 μm apart). Sections were counter stained with DAPI (1 μg/ml) and mounted with Vectashield (Invitrogen). Biodistribution was determined using confocal microscopy with an optical thickness of 1 μm (60× objective). A total of 6 individual images from different regions of the lung per animal/section were analysed and representative images are shown.

For transfection of K18-lacZ mice, siRNA or asDNA were complexed to GL67 and mice were transfected as described above (40 and 160 μg/mouse, respectively). At indicated time-points after transfection lungs were harvested, split in half and processed for βgal mRNA (see below) and protein quantification. The 72 hours control group in the asDNA mRNA graph is missing for technical reasons. For the βgal protein quantification lungs were homogenised in 500 μl Universal Lysis Buffer (Roche), freeze/thawed three times, spun at 10,000 g_av _for 10 min and supernatant was frozen for analysis. βgal protein expression was quantified in lung homogenates using the luminescent βgal Reporter System 3 (BD Biosciences Clontech, Palo Alto, USA) according to manufacturer's recommendations. Total protein was quantified using the DC Protein Assay Kit (BioRad, Herts, UK) according to manufacturer's recommendations and data expressed as pg βgal/mg total protein. Two independent experiments were carried out for each condition.

### *In vivo *transfection of nose and PD measurements

Fluorescently (FITC)-labelled asDNA or siRNA were complexed to GL67 (80 μg/mouse in 100 μl total volume). Mice (BALB/C, female 6–10 weeks) were anaesthetised [one part Hypnorm (Janssen Animal Health, Oxford, UK), one part Hypnovel (Roche, Welwyn Garden City, UK), and two parts water for injection (10 ml/kg)] and placed onto heated boards in the supine position. A fine tip catheter was inserted 5 mm into the nasal cavity and the liposome formulation was slowly perfused (1.3 μl/min) over 75 min using a peristaltic pump. During the procedures the animals were placed at an angle (approximately 45° head down) to prevent aspiration. One or twenty-four hours after transfection animals were culled, the nasal septum removed and fixed over-night in 4% paraformaldehyde. The tissues were processed and paraffin-embedded using standard procedures and 5 μm sections cut (at least 5/mouse approximately 50 μm apart). Sections were counter stained with DAPI (1 μg/ml) and mounted with Vectashield (Molecular Probes). Distribution was determined using confocal microscopy with an optical thickness of 1 μm (60× objective). A total of 6 individual images from different regions of the septum per animal were analysed and representative images are shown.

For transfection with ENaC asODN5, the asODN was complexed to GL67 and the mouse transfected as described above. Twenty-four, 48 and 72 hours after transfection nasal potential difference (PD) was measured as described below, after which the animal was culled and the nasal septum removed for RNA extraction and mRNA quantification.

PD measurements were carried out as previously described [16]. In brief, a fine, double-lumen polyethylene catheter was inserted into the nose. One lumen conveyed perfusate via a peristaltic pump (Pharmacia, Cambridge, UK) at a rate of 21 μl/min, and the other served as an exploring electrode connected via a calomel electrode (Russell pH Ltd., Auchtermuchty, Scotland, UK) to a handheld computer (Psion, London, UK) containing a low-pass signal-averaging filter with a time constant of 0.5 s (Logan Research Ltd., Sussex, UK). A reference electrode was placed subcutaneously in the flank of the mouse and was similarly connected to the computer. The circuit was validated with a measurement of buccal PD prior to insertion of the catheter, acceptable values being 10 to 20 mV. After recording of baseline PD, animals were perfused with a buffer containing amiloride to inhibit sodium absorption via ENaC channels.

### RNA extraction and quantitative RT-PCR

For RNA extraction tissue samples were immediately submerged in RNAlater (Ambion, Huntingdon, UK) after harvesting and stored at or below 4°C until further analysis. Cells were immediately submerged in RLT buffer (Qiagen, Germany) and stored at -80°C. Tissue samples were homogenized in RLT buffer and cell samples were passed through a QiaShredder (Qiagen Ltd, Crawley UK) prior to extraction of total RNA using the RNeasy mini protocol (Qiagen). Levels of mRNA were quantified by real-time quantitative multiplex TaqMan RT-PCR using the ABI Prism 7700 Sequence Detection System and Sequence Detector v1.6.3 software (Applied Biosystems, Warrington, Cheshire, UK). The oligonucleotide primer and fluorogenic probe sequences were designed using Primer Express Software version 1.5 (Applied Biosystems). LacZ mRNA was quantified using forward LacZ primer (5' ATC AGG ATA TGT GGC GGA TGA 3'), reverse LacZ primer (5' CTG ATT TGT GTA GTC GGT TTA TGC A 3'), and fluorogenic LacZ probe (5' FAM- CGG CAT TTT CCG TGA CGT CTC GTT -TAMRA 3'). ENaC mRNA was quantified using forward ENaC primer (5' GAC CTC CAT CAG TAT GAG AAA GGA A 3'), reverse ENaC primer (5' GAC ATC GCT GCC ATT CTC AGT 3'), and fluorogenic ENaC probe (5' VIC- CCT GGA CAG CCT CGG AGG CAA CTA -TAMRA 3'). mCFTR was quantified using forward mCFTR primer (5' TCG TGA TCA CAT CAG AAA TTA TTG ATA AT 3'), reverse mCFTR primer (5' CCA CCT CTC TCA AGT TTT CAA TCA T 3') and fluorogenic mCFTR probe (5' FAM- CGC TCA TTC CCA ACA ATA TGC CTT AAC AGA ATA -TAMRA 3').

RNA was reverse transcribed with TaqMan RT reagents (Applied Biosystems). The RT-reaction mix (5 μl) consisted of 1X TaqMan RT buffer, 5.5 mM MgCl_2_, 500 μM each dNTP, 0.4 U/μl RNase inhibitor, 1.25 U/μl MultiScribe Reverse Transcriptase, 0.4 μM of LacZ or ENaC reverse primer, 0.4 μM of rRNA reverse primer and approximately 50 or 100 ng total RNA for ENaC or LacZ quantification, respectively. Reactions were incubated at 48°C for 30 min followed by 95°C for 5 min. Subsequently, triplicate 25-μl PCRs were performed for each sample. Each 25-μl reaction consisted of 1X TaqMan Universal PCR Mastermix (Applied Biosystems), 300 nM forward primer, 300 nM reverse primer, 100 nM probe, and 5 μl reverse-transcribed template. Reactions were incubated at 50°C for 2 min and then 95°C for 10 min followed by 40 cycles of 95°C for 15 s and 60°C for 1 min. Controls included no-template and no-reverse transcriptase reactions in which total RNA or MultiScribe reverse transcriptase and RNase inhibitor were omitted from the reverse transcriptase reaction, respectively. Relative levels of ENaC- or LacZ-specific mRNA were determined using the ΔΔ*C*_T _method [17]. In this study the amount of target LacZ or ENaC was normalized to ENaC or murine CFTR, respectively (endogenous reference) and expressed relative to an arbitrary calibrator sample that was used throughout the study. The calibrators used were RNA derived from a K18-LacZ transgenic mouse lung for LacZ quantification and naïve mouse lung for ENaC quantification.

### Statistical Analysis

Values are expressed as the mean ± SEM for convenience or dot plots plus mean. n refers to the number of animals or tissue culture samples used. Data were compared using ANOVA plus post hoc analysis or independent sample t-test where appropriate or paired sample test for PD measurements. The null hypothesis was rejected at p < 0.05.

## Results

### Assessment of the physical characteristics of GL67-complexed small nucleic acids

**Figure 1:**
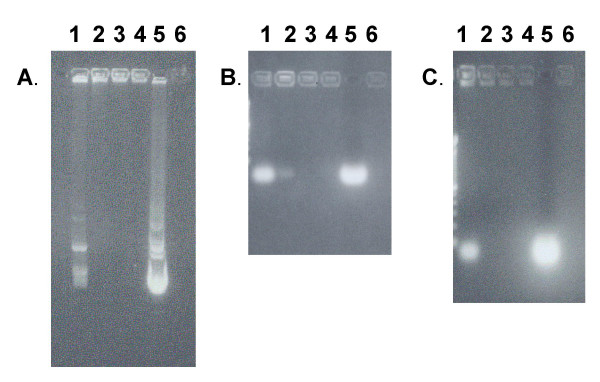
**Gel retardation of Genzyme lipid 67 (GL67)-complexed plasmid DNA, siRNA and asODN **. Plasmid DNA (A), siRNA (B) or asODN (C) were complexed to GL67 at different lipid:nucleic acid molar ratios and complexes were separated on agarose gels. (Lane 1 = 0.25:1, 2 = 0.5:1, 3 = 0.75:1, 4 = 1:1 lipid: nucleic acid molar ratios, 5 = no lipid control, 6 = empty well).

The characteristics of plasmid DNA(pDNA)-Genzyme Lipid 67 (GL67) complexes have been described [18]. To compare these with small nucleic acid molecules asODNAs and siRNAs we generated lipoplexes [18] using a range of lipid:nucleotide molar ratios (0.25:1 to 1:1). Agarose gels (Figure 1) showed that at the 0.25:1 ratio, which is the most efficient ratio for lung gene transfer [18], approximately 75% of the nucleotides (siRNA, asODN or pDNA) were incorporated within lipoplexes In all cases, increasing the lipid:nucleotide ratio further increased the amount of complexed nucleic acid. Light scatter analysis was used to determine the size of the lipoplexes, which were 294 ± 17, 369 ± 24 and 682 ± 235 nm for siRNA, asODN and pDNA, respectively at the 0.25:1 molar ratio and did not change at the 0.75:1 ratio (data not shown, n = 4/group in 2 independent experiments).

### Stability of siRNAs and asODNs following exposure exhaled breath condensate (EBC)

**Figure 2:**
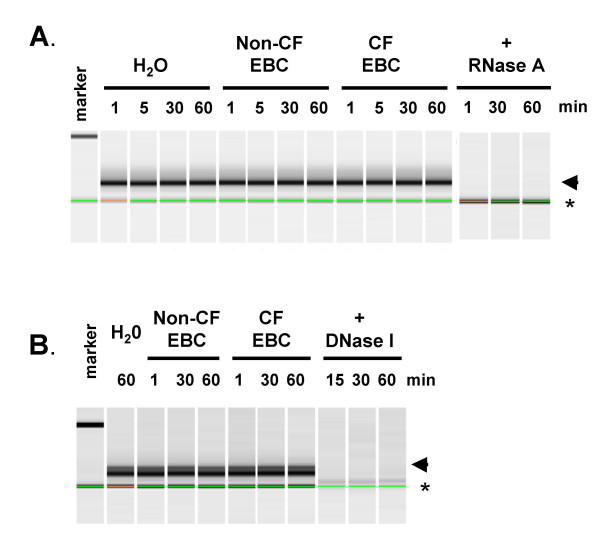
**Stability of siRNA and asODN in exhaled breath condensate (EBC)**. siRNA (A) or asODN (B) were incubated for one to 60 min in water or EBC from CF and non-CF individuals. Oligonucleotide integrity was assessed using the Agilent Bioanalyser 2100 system. In control reactions siRNA and asODN were incubated with RNase A and DNase I, respectively, for one to 60 min. Arrowhead indicates oligonucleotides. Asterisk indicates lane marker.

Nucleic acids are prone to nuclease degradation. The stability of phosphorothioated asODN, in a variety of body fluids is well described, but neither the stability of asODNs or siRNAs has been studied in airway surface liquid (ASL), a potential barrier to transfection of the airway epithelium. ASL is difficult to collect, and we therefore used exhaled breath condensate (EBC) as a surrogate. Uncomplexed siRNA and asDNA nucleic acids were incubated in CF (n = 2) and non-CF (n = 2) EBC samples, or water, for 1–60 min. No evidence of nucleic acid degradation was observed (Figure 2). In control experiments siRNAs or asODNs were incubated with either RNase A or DNase I, respectively, for 1 to 60 min. In both cases complete degradation of the siRNA and asODN was seen (Figure 2).

### Intracellular location of siRNAs and asODNs *in vitro*

**Figure 3:**
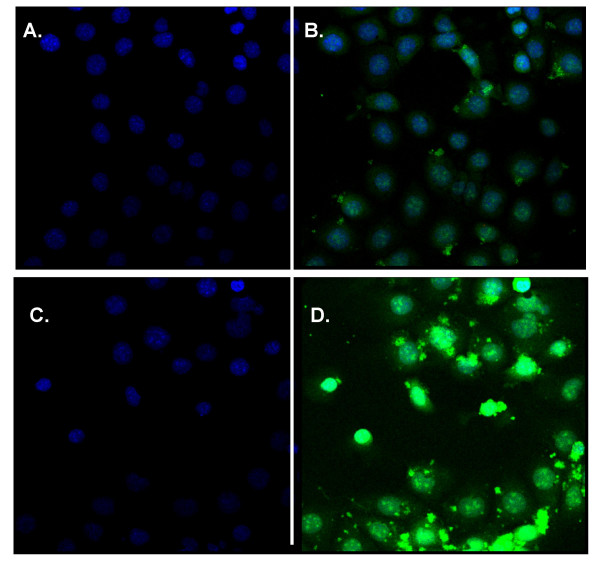
**Distribution of FITC-labelled asODN in M1 cells *in vitro***. M1 cells were transfected with Lipofectamine 2000-complexed FITC-labelled asODN. At indicated time-points after transfection cells were harvested and processed for confocal microscopy. Nuclei were stained with DAPI and are shown in blue (left panel), FITC signal is shown in green (right panel). A and B show biodistribution 30 min after transfection, C and D show biodistribution 24 hrs after transfection.

**Figure 4:**
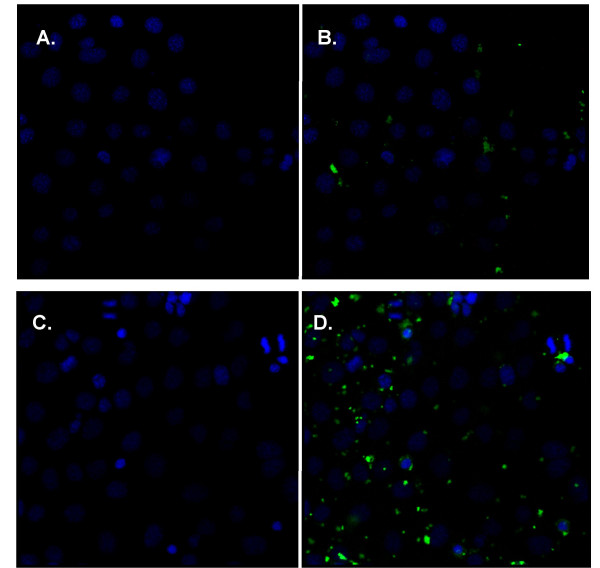
**Distribution of FITC-labelled siRNA in M1 cells *in vitro***. M1 cells were transfected with Lipofectamine 2000-complexed FITC-labelled siRNA. At indicated time-points after transfection cells were harvested and processed for confocal microscopy. Nuclei were stained with DAPI and are shown in blue (left panel), FITC signal is shown in green (right panel). A and B show biodistribution 30 min after transfection, C and D show biodistribution 24 hrs after transfection.

M1 cells, a murine kidney cell line, express ENaC [19] and are, therefore, suitable for screening anti-ENaC siRNA and asODN sequences (see below). However, these cells have not been routinely used for transfection experiments. Here, we first determined transfection efficiency using FITC-labelled siRNAs and asODNs complexed to Lipofectamine 2000, one of the most efficient liposomes for *in vitro *nucleic acid gene transfer. AsODNs rapidly (as early as 30 min after transfection, Figure 3A+B) accumulated in the nucleus of transfected cells, whereas siRNA was only detectable in the cytoplasm (Figure 4A–D, n = 3 wells/time point). Twenty-four hours after transfection approximately 80–90% of cells were transfected with either molecule (Figure 3C and 3D) and the overall distribution remained unchanged, with asODNs accumulating in the nuclei and siRNA in the cytoplasm. In control experiments we also transfected cells with double-stranded DNA oligonucleotides (dsODN); nuclear accumulation was similar to single-stranded asODN (data not shown). Thus interestingly, the intracellular localisation of siRNA and ODN appear to be consistent with their presumed sites of action.

### Distribution of siRNA- and asODN in the murine lung *in vivo*

**Figure 5:**
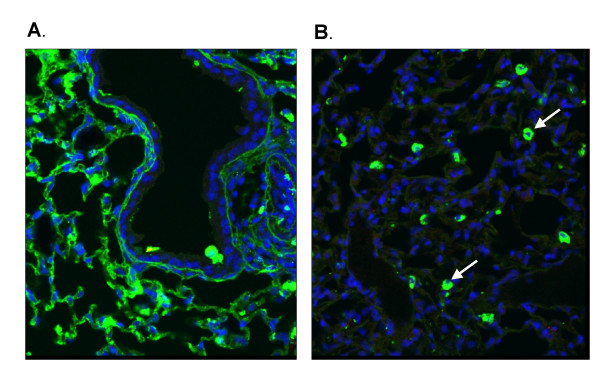
**Distribution of FITC-labelled siRNA and asODN in mouse lung**. FITC-labelled asODN (a) and siRNA (b) (160 μg/mouse) were complexed to GL67 and "sniffed" into mouse lung. One or 24 hours after transfection the lungs were paraffin-embedded and processed for confocal microscopy. Nuclei are shown in blue, FITC signal is shown in green. Arrow indicates alveolar macrophage.

Genzyme Lipid (GL67) has been optimised for gene transfer to the airway epithelium and has been used in CF gene therapy trials [20,21] and was, therefore, an obvious choice for delivering asODN and siRNA to the airways. We administered FITC-labelled siRNA and asODN (160 μg/mouse) complexed to GL67 to the mouse lung using a standard intranasal instillation protocol to determine distribution (n = 3/group). Interestingly, 24 hours after transfection the distribution of the two molecules was very different. Abundant asODN signal was visible in the cytoplasm of alveolar epithelial cells (Figure 5A), with only sporadic signal in the airway epithelium whereas siRNA could only be detected in alveolar macrophages (Figure 5B). For both siRNA and asODN, there was no difference in staining pattern one hour (data not shown) and 24 hours after transfection.

### Efficacy of siRNA- and asODN-mediated gene silencing in the murine lung *in vivo*

Although FITC-labelled nucleic acids is an informative way to assess bio-distribution, tracking low levels of transfection in airway epithelial cells may have been below the detection limit of this assay. To address this potential problem, we studied K18-lacZ transgenic mice, which express β-galactosidase (β-gal) in airway epithelial cells (20) as a functional read-out of transfection efficiency. We first designed and tested 10 *lacZ *siRNA and 5 *lac Z *asODN (see Table 1 for sequences) in NIH-3T3 cells stably expressing LacZ. Three out of 10 *lacZ *siRNA reduced βgal expression by >50% relative to control levels (Z4: 919 ± 315 pg β-gal/mg protein; Z5: 1135 ± 194 pg β-gal/mg protein, Z7: 976 ± 310 pg β-gal/mg protein, control siRNAs: CAT: 2791 ± 306 pg β-gal/mg protein, GFP: 2896 ± 385 pg β-gal/mg protein); Z7 was chosen for further *in vivo *studies. All five *lacZ *asODN reduced expression between 40–60% relative to control asODN assayed 48 hrs after transfection. The most effective asODN (as4) reduced lacZ expression from 3133+/-346 pg βgal/mg protein in controls to 1044+/-142 pg/mg in asODN treated cells (p < 0.05) and this asODN was used in subsequent *in vivo *experiments.

**Table 1:** siRNA and asDNA used in this study

**Name**	**Sequence (from 5' to 3')**	
Antisense oligonucleotides		
FITC-asDNA	UACGATGCTGCTAGCUAGUA	
LacZ as 1	AUCAUCATTAAAGCGAGUGG	
LacZ as 2	AUGGAAACCGTCGATAUUCA	
LacZ as 3	GGAAGGATCGACAGAUUUGA	
LacZ as 4	AACAGGTATTCGCTGGUCAC	
LacZ as 5	CCAUGCCGTGGGTTTCAAUA	
Control 1	UACGATGCTGCTAGCUGUAC	
Control 2	UCGAUGTAGCTAGCTAUGUC	
αENaC asODN 1	GAAUGGAGGAGGATGUCAGA	
αENaC asODN 2	ACCGUGGATGGTGGTAUUGU	
αENaC asODN 3	GUUGAAACGACAGGTAAAGA	
αENaC asODN 4	GUGGAAGATGTGCTGAAGUG	
αENaC asODN 5	UUCUGGTTGCACAGTUGGAA	
Synthetic small interfering RNAs		
LacZ siRNA 1	Sense Z1 Antisense Z1	5'PO_4 _r(acc cug gcg uua ccc aac uua a)3'OH 5'PO_4 _r(aag uug ggu aac gcc agg guu u)3'OH
LacZ siRNA 2	Sense Z2 Antisense Z2	5'PO_4 _r(gcu ggc ugg agu gcg auc uu)3'OH 5'PO_4 _r(gau cgc acu cca gcc agc uu)3'OH
LacZ siRNA 3	Sense Z3 Antisense Z3	5'PO_4_r(ccu auc cca uua cgg uca auc c)3'OH 5'PO_4_r(auu gac cgu aau ggg aua gg)3'OH
LacZ siRNA 4	Sense Z4 Antisense Z4	5'PO_4 _r(ccg acu aca caa auc agc gau u)3'OH 5'PO_4 _r(ucg cug auu ugu gua guc ggu u)3'OH
LacZ siRNA 5	Sense Z5 Antisense Z5	5'PO_4 _r(guu cag aug ugc ggc gag uu)3'OH3 5'PO_4 _r(cuc gcc gca cau cug aac uu)3'OH
LacZ siRNA 6	Sense Z6 Antisense Z6	5'PO_4 _r(cuu uaa cgc cgu gcg cug uu)3'OH 5'PO_4 _r(cag cgc acg gcg uua aag uu)3'OH
LacZ siRNA 7	Sense Z7 Antisense Z7	5'PO_4 _r(gcc aau auu gaa acc cac gg)3'OH 5'PO_4 _r(gug ggu uuc aau auu ggc uu)3'OH
LacZ siRNA 8	Sense Z8 Antisense Z8	5'PO_4 _r(cug ugc cga aau ggu cca uca a)3'OH 5'PO_4 _r(gau gga cca uuu cgg cac agc c)3'OH
LacZ siRNA 9	Sense Z9 Antisense Z9	5'PO_4 _r(gca aaa cac cag cag cag uu)3'OH 5'PO_4 _r(cug cug cug gug uuu ugc uu)3'OH
LacZ siRNA 10	Sense Z10 Antisense Z10	5'PO_4 _r(gug acc agc gaa uac cug uu)3'OH 5'PO_4 _r(cag gua uuc gcu ggu cac uu)3'OH
**Control siRNAs**		
CAT	sense antisense	5'PO_4 _r(gag uga aua cca cga cga uuu c) 3' OH 5'PO_4 _r(aau cgu cgu ggu auu cac ucc a) 3' OH
FITC-CAT	sense antisense	5'PO_4 _r(gag uga aua cca cga cga uuu c) 3' Fluorescein 5'PO_4 _r(aau cgu cgu ggu auu cac ucc a) 3' OH
GFP	sense Antisense	5'PO_4 _r(gca agc uga ccc uga agu uca u) 3' OH 5'PO_4 _r(gaa cuu cag ggu cag cuu gcc g) 3' OH

**Figure 6:**
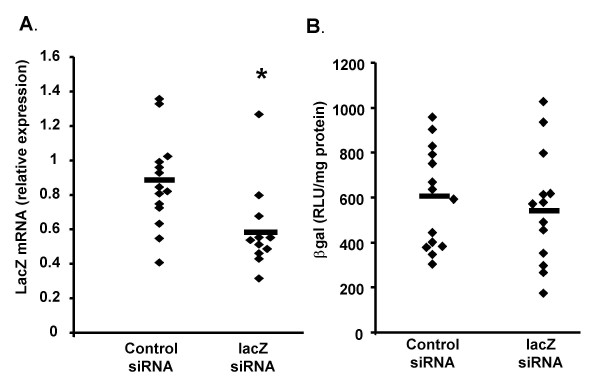
***In vivo *lung transfection of K18-lacZ with lacZ siRNA**. LacZ siRNA (Z7) or control siRNA was complexed to GL67 (40 μg/mouse), placed as a bolus (100 μl) onto the nostrils of anaesthetised mice and "sniffed" into the lung. Forty-eight hours after transfection the lungs were harvested and βgal mRNA (A) and protein expression (B) were quantified. Each diamond represents an individual animal. The mean per group is indicated as a horizontal bar. * indicates p < 0.05 when compared to control group.

The *lacZ *(Z7) siRNA was complexed to GL67 and administered by intranasal instillation to the mouse lung. Forty-eight hours later lungs were harvested, divided into two parts and used for mRNA quantification (quantitative RT-PCR) and βgal protein quantification (Figure 6). In animals treated with *lacZ *siRNA βgal mRNA was reduced by approximately 33% when compared to controls (*lacZ *siRNA: 0.58 ± 0.07, controls siRNA: 0.87 ± 0.07 relative *lacZ *mRNA expression, n = 12–14/group, p < 0.01). However, there was no significant change in βgal protein expression.

**Figure 7:**
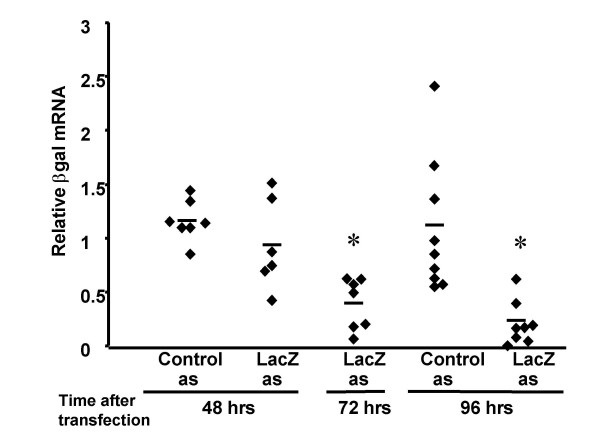
**βgal mRNA *in vivo *lung transfection of K18-lacZ with lacZ asODN**. LacZ asODN (as4) or a control ODN was complexed to GL67 (160 μg/mouse), placed as a bolus (100 μl) onto the nostrils of anaesthetised mice and "sniffed" into the lung. Forty-eight to 96 hours after transfection the lungs were harvested and β-gal mRNA was quantified. Each diamond represents an individual animal. The mean per group is indicated as a horizontal bar. * indicates p < 0.05 when compared to control group.

**Figure 8:**
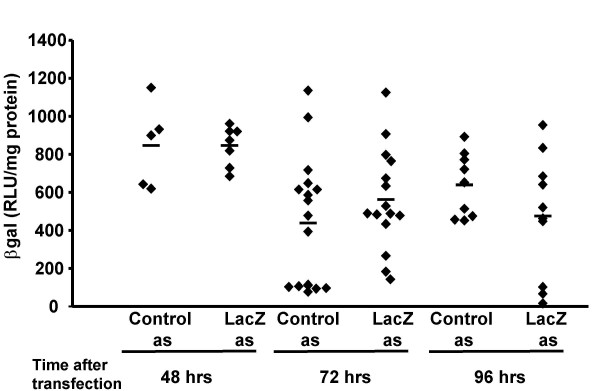
**βgal protein after *in vivo *lung transfection of K18-lacZ with lacZ asODN**. LacZ asODN (as4) or a control ODN was complexed to GL67 (160 μg/mouse), placed as a bolus (100 μl) onto the nostrils of anaesthetised mice and "sniffed" into the lung. Forty-eight to 96 hours after transfection the lungs were harvested β-gal protein expression was quantified. Each diamond represents an individual animal. The mean per group is indicated as a horizontal bar. * indicates p < 0.05 when compared to control group.

*LacZ *asODN was also complexed to GL67 and administered by intranasal instillation. Gene expression was analysed 48, 72 and 96 hours after administration. There was no decrease in *lacZ *mRNA at 48 hours, but 72 and 96 hours after transfection *lacZ *mRNA was significantly (p < 0.01) reduced by >60% (Figure 7) when compared to controls (n = 6–9/group). Similar to the siRNA there was no decrease in lacZ protein expression at any time-point studied (Figure 8). It is worth noting that these studies were only powered (80% power at p < 0.05) to detect a decrease in βgal protein expression of at least 60%.

### Distribution of asODN and siRNA in the murine nose in vivo

Inefficient transfection of airway epithelial cells may be a key limiting factor in achieving meaningful levels of down-regulation of gene expression *in vivo*. The short contact time between airway epithelial cells and the administered complexes, as well as their pooling in alveolar region following the intranasal method likely contributes to this inefficiency. The murine nasal epithelium has a cell composition similar to that of the lower airways, and allows increased contact time by slow perfusion of the complexes directly onto the nasal epithelium via a catheter. This route of administration also allowed us to make functional measurements of *ENaC *silencing, via transepithelial potential difference measurements which can easily be undertaken in the murine nose.

**Figure 9:**
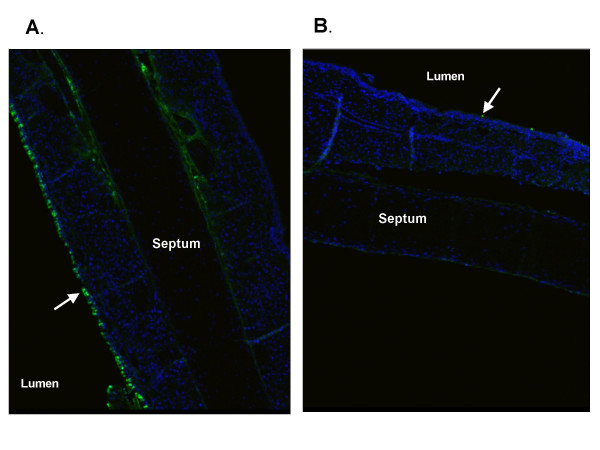
**Distribution of FITC-labelled asODN in the mouse nose *in vivo***. FITC-labelled asODN (80 μg/mouse) were complexed with GL67 and perfused into the nasal cavity. Twenty-four hours later the nasal septum was extracted, paraffin-embedded and processed for confocal microscopy. Nuclei are shown in blue, FITC signal is shown in green. Arrows indicate the surface epithelium. A and B are images from two different animals showing the highest (A) and lowest (B) levels of uptake seen.

To assess uptake efficiency in this region we perfused animals with FITC-labelled asODN or siRNAs complexed to GL67 (80 μg/mouse) and harvested tissues one or 24 hours after delivery (n = 6). No uptake of siRNA into nasal epithelial cells was seen at any time-point (data not shown) and, therefore, no further studies were undertaken with siRNA. For asODN the efficiency of uptake varied greatly from animal to animal. Figure 9A+B shows representative images from two treated animals covering the range of uptake seen. In general efficiency of uptake was low, with most animals (5 out of 6 mice) having only a few (<10/section) FITC-positive cells. There was no difference in staining pattern one hour (data not shown) and 24 hours after transfection.

### Efficacy of anti ENaC asODN in the murine nose *in vivo*

**Figure 10:**
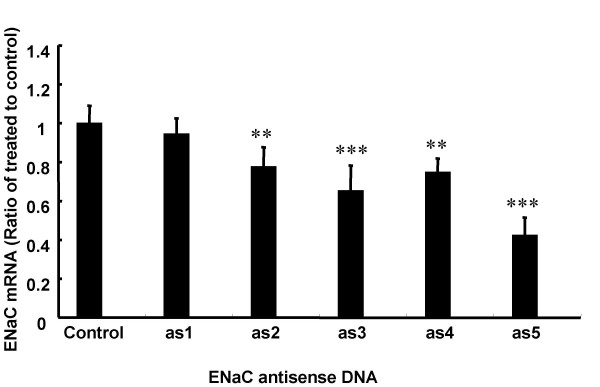
***In vitro *screening of ENaC asODN in M1 cells**. M1 cells were transfected with 5 different αENaC asODN and a control ODN complexed to Lipofectamine 2000. Forty-eight hours after transfection mRNA was extracted and αENaC mRNA was quantified. Data are presented as mean +/- SEM. ** indicates p < 0.01 and *** = p < 0.001 when compared to control, n = 6/group.

To assess the efficacy of asODN against ENaC we first designed five *ENaC *asODN against the murine α subunit (αENaC asODN) and transfected into M1 cells using standard transfection conditions. Forty-eight hours after transfection cells were harvested and αENaC mRNA was quantified (Figure 10). The relative degree of αENaC mRNA reduction varied with asODNs and ranged from 5 to 60% (n = 6/group). Subsequent *in vivo *experiments were carried out using αENaC asODN 5.

**Figure 11:**
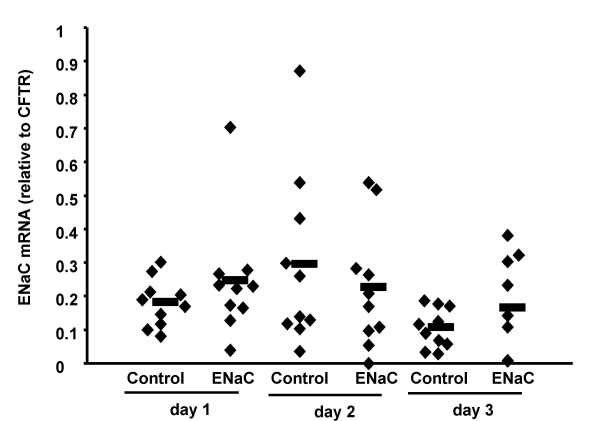
**αENaC mRNA in nasal tissue after *in vivo *transfection with ENaC asODN **. αENaC asODN (as5) or a control ODN was complexed to GL67 (80 μg/mouse) and slowly perfused onto the nasal epithelium of mice via a thin catheter. At indicated times after transfection nasal potential difference measurements were carried out (for results see Table 2) and the nasal tissues was harvested for mRNA extraction and quantification of αENaC mRNA. Each diamond represents an individual animal. The mean per group is indicated as a horizontal bar.

Transepithelial potential difference measurements (PD) have been widely used to measure ion transport across murine nasal epithelium (21). A decrease in ENaC function would result in (a) a drop in baseline PD, because sodium transport is the main component of basal ion transport in the nasal mucosa and (b) a reduced effect of the ENaC blocker amiloride. We complexed αENaC asODN4 to GL67 (80 μg/mouse) and perfused these complexes into the mouse nose over 75 min. Twenty-four, 48 or 72 hours after administration basal PD and the effect of amiloride were measured in individual cohorts of animals (n = 10/group). After PD measurements all animals were culled and quantitative RT-PCR was carried out on nasal mRNA. We did not detect changes in baseline PD or the effect of amiloride at any time-point (Table 2), nor any reduction in αENaC mRNA (Figure 11). Thus, despite ensuring prolonged contact time of asODN/GL67 complexes with the nasal epithelial cells, transfection efficiency was variable and likely insufficient to reduce ENaC expression.

**Table 2:** Nasal potential difference measurements in mouse nasal epithelium after perfusion with ENaC antisense DNA

**Baseline PD (mV)**					**Amiloride effect (ΔmV)**			
**Day**	**Control asODN**		**ENaC asODN**		**Control asODN**		**ENaC asODN**	
	**Pre**	**Post**	**Pre**	**Post**	**Pre**	**Post**	**Pre**	**Post**

1	15.7 (1.8)	16.3 (1.6)	15.1 (1.8)	20.4 (2.1)	-14.3 (1.8)	-9.1 (1.3)	-14.6 (1.9)	-9.7 (1.4)
2	16.9 (2.3)	17.2 (1.7)	16.0 (1.2)	16.3 (2.0)	-15.7 (2.3)	-13.7 (1.6)	-14.8 (1.4)	-12.1 (1.3)
3	16.8 (1.9)	17.0 (1.9)	18.0 (1.9)	16.1 (0.9)	-12.1 (1.5)	-13.0 (2.0)	-14.8 (1.4)	-12.6 (1.3)

n = 8–10/group. Data are expressed as mean (SEM)

PD = nasal potential difference

Pre = measurements at least 7 to 10 days before administration of control or ENaC asODNs

Post = measurements at the indicated time-points after administration of control or ENaC asODNs.

## Discussion

We have assessed the transfection efficiency and efficacy of small (approximately 20 bp) nucleic acids (siRNA and antisense DNA) complexed to GL67 in conducting airway epithelium *in vivo*. In general, uptake of small oligonucleotides was inefficient and although mRNA levels were reduced, we could not reduce protein levels or ENaC function. This study suggests that although siRNAs and asODNs can be developed to inhibit gene expression in culture systems and certain organs *in vivo*, barriers to nucleic acid transfer in airway epithelial cells seen with large DNA molecules also affect the efficiency of *in vivo *uptake of small nucleic acid molecules.

It is unlikely that alteration in nucleic acid size alone will result in improved airway nucleic acid transfer. We suggest, that the asODN and siRNA-based strategies may not be successful in conducting airway epithelium, until nucleic acid transfer is optimised further.

Interestingly, the intracellular distribution of asODN and siRNA following *in vitro *transfection into epithelial cells was very different, with asODN-lipoplexes accumulating rapidly in the nucleus while siRNA-lipoplexes were seen as a diffuse staining in the cytoplasm. For asODN this distribution was independent of nucleotide sequence, and has also recently been seen in other cell lines, such as A549 and HEK293 cells (Chris Kitson, GlaxoSmithKline, personal communication). To the best of our knowledge, we show, for the first time, that the respective localisations *in vitro *are consistent with the presumed sites of action of each of the molecules in inhibiting gene expression [23].

Various cationic lipids and polymers have been assessed for gene transfer to the airways, but information on a "best buy" non-viral transfer agents for small nucleic acid delivery does not currently exist. In our hands, GL67 has been most efficient for airway gene transfer and we have assessed its safety in phase 1 trials in normal volunteers and CF patients [20,21]. Here, we assessed the efficacy for GL67 to delivery asODN and siRNA to the airways. *In vivo*, siRNA-lipoplexes were found predominantly associated with alveolar macrophages, whereas asODNs were, as has been reported previously, associated with pneumocytes. The reasons for this difference in cellular distribution is currently unclear, but may relate to differences in RNA versus DNA-lipoplex properties. Small differences in overall complex structure may for example alter the surface charge slightly, which in turn may affect cell uptake. Zhang and co-workers recently described administration of "naked" biotin-labelled siRNA to the lung and reported diffuse staining in airways and parenchyma [24]. We were unable to detect any signal after administration of "naked" FITC-labelled siRNA in the lung (data not shown). This may in part reflect different detection limits of these methods (FITC versus biotin-streptavidin) in the lung, possibly due to high auto-fluorescence of lung tissue. We speculate that the differences in cellular distribution of asODN *in vitro *(mainly nuclear) and *in vivo *(mainly cytoplasmic) may be due to differences in the proliferation status of the transfected cells. M1 cells replicate rapidly, whereas the majority of pneumocytes are terminally differentiated non-dividing cells. The nuclear membrane is likely to present a significant barrier to ODN uptake *in vivo*.

The inefficient and variable degree of transfection of the airway epithelium was also reflected in the experiments testing inhibition of gene expression. In the airway epithelium of K18-lacZ transgenic mice the administration of lacZ asODN significantly reduced βgal mRNA 72 and 96, but not 48 hours, after transfection. In contrast, following *lacZ *siRNA transfection *lacZ *mRNA levels were significantly reduced 48 hours after transfection. The delayed response of asODNSs, may be due to the different sites and modes of action. In contrast to mRNA there was no reduction in βgal protein at any time point tested. There are a number of possible explanations. The predicted half-life of βgal protein is 20 hours. Harvesting lungs 48 hours after transfection should, therefore, have allowed a reduction in protein expression to be seen. However, the half-life of βgal protein *in vivo *may be longer than 20 hours, reducing the likelihood of detecting a significant change in protein levels during the time course of these experiments. In addition, we cannot exclude a non-linear response between protein amounts and enzymatic activity, which may require a substantial reduction in protein before any changes in enzymatic activity can be seen. Finally, even with the relatively large numbers studied, we were only powered to detect a protein reduction of 60% or more.

Recently several studies described the successful use of siRNA to inhibit pulmonary influenza virus and respiratory syncytial virus infections [25-27] using intravenous (IV) or intranasal (IN) administration of plasmid DNA encoding the siRNA or IV plus IN administration of siRNA. These studies did not specifically assess the effects of RNA interference in difficult to transfect conducting airway epithelial cells, but looked at global anti-viral responses in the lung. To the best of our knowledge efficacy of RNAi in airway epithelium has not been studied yet.

We tried to improve transfection efficiency by prolonging contact time with the airway epithelial cells. For practical reasons this necessitated a change to the nose. Despite this, we could not detect any intracellular fluorescent signal after administration of FITC-labelled siRNA/GL67 complexes and only a low and highly variable signal of asODN/GL67 complexes. Importantly, the overall transfection efficiency of nasal and lung airway epithelial cells was identical, despite anatomical differences, lending credit to using the nasal epithelium as a surrogate for airway nucleic acid transfer.

Despite using antisense ODNs that reduced αENaC mRNA by up to 60% *in vitro*, results consistent with other gene targets in our hands, these did not reduce ENaC mRNA or protein function in murine nasal epithelium *in vivo*. The reported half-life of the ENaC protein varies and appears to be cell-type specific, but is of the order of 40–120 min in cultured cells [28] and 3–4 hours in Xenopus oocytes [29]. We know of no data for the murine airways. Thus, although the stability of the protein may play a role, we suggest that poor transfection is more likely the reason for the lack of effect.

The efficiency of siRNA and antisense inhibition is sequence dependent and though some attempts have been made to develop computer-assisted design of these types of molecules extensive biological testing is still required. The *LacZ *siRNAs described in this study were developed using first generation design guidelines that focused on the overall nucleotide composition and distribution (50:50 AU:GC ratio, with approximately equal distribution of pyrimidines and purines throughout the molecule). These siRNAs were analyzed prior to the development of second-generation guidelines that focus on more mechanistically relevant features of siRNAs, particularly a nucleotide composition that favours asymmetrically loading of siRNAs into the ribonucleoprotein complex that mediates RNAi. Using the first generation guidelines 3 out of 10 siRNAs mediated a significant degree of silencing. In our experience this proportion of effective siRNAs and asODNs reflected the success rate seen for the design of siRNAs against most transcripts using first generation guidelines.

A key development in improving small nucleic acid delivery *in vivo*, particularly of asODNs, has been modification of the molecules or their delivery agent to improve their stability in biological fluids. Neither asODN nor siRNA stability has been studied in the context of the extracellular lung environment in humans, though Templin *et al *nebulised asODN into the murine lung and reported a half-life of >20 hours [30]. The airway surface liquid (ASL) lining the conducting airways is important for host defence and contains numerous anti-microbial peptides [31]. Collection of unperturbed ASL in human lung is impossible and broncho-alveolar lavages (using comparatively large volumes) lead to dilution of ASL components. Although exhaled breath condensate (EBC), which can be collected non-invasively and consists of condensed water and microdroplets, containing volatile and non-volatile compounds representing ASL from the lower respiratory tract [32,33], may not mimic all aspects of ASL, it goes some way towards assessing the stability of small nucleic acid in the human lung. Importantly, stability of asODNs and siRNAs in EBC from inflamed lungs of CF patients was similar to non-CF patients. These data support previously published stability data for second-generation asODN, [where one of the non-bridging oxygen atoms in the phosphodiester bond is replaced with sulphur (phosporothioation)], and the addition of 2' O-methylated RNA residues increase nuclease resistance, and also support the apparent nuclease stability of siRNA even in the absence of chemical modification. We did not assess stability of GL67/siRNA or asODN complexes, because it has previously been shown that lipid complexing further increases nuclease stability of oligonucleotides [34].

## Conclusion

In summary, gene silencing targeted to conducting airway epithelial cells is currently inefficient, likely due to the relatively poor transfection efficiency of airway cells with siRNA and asODN lipid complexes. Techniques for small nucleic acid transfection are needed if the *in vitro *promise of these molecules is to be translated to airway epithelial cells *in vivo*.

## Declaration of competing interests

The author(s) declare that they have no competing interests.

## Authors' contributions

UG, CK, NJC and EWFWA conceived the study and wrote the manuscript. SEG, RF, CS, LS, HP, RLS, DRG, SCH, ME carried out all in vitro and in vivo work and molecular analysis and contributed to the design of the study. VO and GM carried out the stability testing in EBC, MG participated in the design of the study. YHC and JH provided the K18 mice, RKS and SC provided GL67. All authors read and approved the manuscript.
